# Cranial radiation disrupts dopaminergic signaling and connectivity in the mammalian brain

**DOI:** 10.1186/s40478-025-01976-3

**Published:** 2025-03-13

**Authors:** Die Zhang, Riya Thomas, Thanh Thai Lam, Ines Veselinovic, David R. Grosshans

**Affiliations:** Departments of Radiation Oncology and Experimental Radiation Oncology, Unit 97, The University of Texas MD Anderson Cancer Center, 1515 Holcombe Blvd, 77030-4009 Houston, TX USA

**Keywords:** Cognitive impairment, Cranial radiation, Mesocortical pathway, Dopamine

## Abstract

Cognitive impairment is a common and challenging side effect of cranial radiation therapy for brain tumors, though its precise mechanisms remain unclear. The mesocortical dopaminergic pathway, known to play a key role in cognitive function, is implicated in several neuropsychiatric disorders, yet its involvement in radiation-induced cognitive dysfunction is unexplored. Here, with using in vivo multi-electrode array recordings of both anesthetized and free-moving rats to monitor the firing activities of dopamine neurons in the ventral tegmental area (VTA) and local field potentials in both the prefrontal cortex (PFC) and VTA, as well as the immunofluorescence assays and western blotting, we report that cranial irradiation transiently altered VTA dopamine neuron firing patterns without affecting overall firing rates and led to sustained reductions in both “awake” and total dopamine neuron density. Additionally, radiation exposure impaired D2 receptor function and disrupted connectivity between the PFC and VTA. These multifaceted disruptions in the mesocortical dopamine signaling may underlie the development of radiation-induced cognitive dysfunction. These findings pave the way for novel research to prevent or reverse radiation-induced injury, ultimately improving the quality of life for brain tumor survivors.

**Supplementary Information:**

The online version contains supplementary material available at 10.1186/s40478-025-01976-3.

## Introduction

For patients with brain tumors, treatment goals include improved survival but also maintenance of quality of life. Although radiation therapy (RT) has a crucial role in the treatment of brain tumors, it is associated with cognitive decline. This decline often manifests as impairments in memory, attention, and executive functioning, with the severity ranging from mild to significant, depending on factors such as radiation dose, treatment volume, and time post-therapy [1]. This is particularly problematic for children with primary brain tumors, for whom survival rates may surpass 70% and the successful treatment of tumors comes at the cost of enduring cognitive decline [2, 3]. This decline, affecting essential cognitive domains like learning, memory, attention, and decision-making, poses a significant, negative, long-term impact on the quality of life for cancer survivors. The consequences extend beyond the individual, placing strain on caregivers, family members, and society as a whole [2, 4]. 

Although traditional theories attribute RT-induced cognitive impairments to the loss of neuronal stem cells in the hippocampus [5, 6], emerging evidence challenges this simplistic hypothesis. For instance, contrary to the prevailing belief, very limited neurogenesis has been detected in the human adult brain [7], and drugs proven to be effective in preventing cognitive decline in animal studies may not necessarily protect against neurogenesis-related deficits [8]. A large randomized clinical trial conducted in adult patients with brain metastases found that, despite acknowledging that cognitive function failure in this diverse group could stem from various factors, including the progression of the metastases themselves, cognitive failure still occurred in 60% of patients following hippocampal-sparing whole-brain radiation therapy [9]. In addition, a recently clinical trial has explored the concept of “memory-avoidance” in whole brain radiation therapy, focusing on sparing critical brain structure such as the amygdala, corpus callosum, and fornix alongside the hippocampus, to preserve cognitive function [10]. Our own pre-clinical work has also identified the pre-frontal cortex as critical structure in the development of RT-induced cognitive dysfunction [11, 12]. These findings highlight a complex interplay of factors contributing to RT-induced cognitive impairments. Beyond the loss of neuronal stem cells in the hippocampus, altered functioning of neurons that survive as well as impaired network activity in other regions of the brain emerge as critical contributors. A nuanced understanding of these and other factors is crucial for developing targeted interventions that address the multifaceted nature of cognitive decline associated with RT.

The mesocortical system, a neural circuitry of paramount importance, is crucial in the maintenance of cognitive functions. Central to this system are the dopaminergic pathways that originate in the ventral tegmental area (VTA) and project to the prefrontal cortex (PFC). These pathways coordinate a range of high-level cognitive functions that involve executive processes or cognitive controls such as attention, planning, problem solving, and decision making [13]. The intricate communication between the VTA and the PFC, facilitated by the neurotransmitter dopamine, form the core of our ability to think, learn, plan, and adapt to new information. However, the susceptibility of this system to various conditions has become increasingly evident. Dysfunctions in the dopaminergic system within the mesocortical network have been implicated in a range of brain diseases associated with cognitive impairments, including Parkinson disease [14, 15, 16], Alzheimer disease [17, 18, 19], attention deficit hyperactivity disorder [20, 21, 22], and schizophrenia [23, 24, 25]. However, to our knowledge, little is known of the effects of radiation on the functionality of dopaminergic signaling. Such knowledge may pave the way to more targeted interventions to prevent or even reverse cognitive dysfunction.

Research conducted in our laboratory has delved into the effects of radiation on neuronal functions beyond the hippocampus, with a particular focus on the PFC [11, 12]. Here we extended our investigation to the mesocortical system, probing its role in radiation-induced cognitive impairments. By using electrophysiological, behavioral, biochemical, and immunohistochemical methods, we provide direct evidence, for the first time, that cranial radiation alters dopamine neuron density and firing activity, dopamine receptor expression and function, and leads to a prolonged depression of the functional coupling between VTA and PFC. Understanding the critical role of the mesocortical dopaminergic pathways in maintaining cognitive functions not only deepens our understanding of cognitive processes but also offers promising avenues for the development of interventions and therapeutic strategies aimed at mitigating cognitive decline in patients undergoing radiation treatments.

## Materials and methods

The Institutional Animal Care and Use Committee of The University of Texas MD Anderson Cancer Center approved all procedures in accordance with federal guidelines. All data analyses were conducted in a blinded manner. The researchers responsible for processing and analyzing the data were provided only with sample numbers or raw data sets, without any information regarding the specific animal identities or treatment groups. This blinding protocol was implemented to eliminate potential bias and ensure the objectivity and reliability of the results.

### Animal care and Preparation

10-week-old Sprague Dawley rats from Harlan Laboratory were used in all experiments. To avoid the influence of reproductive cycles and hormone fluctuations on neuronal activities, only male rats were used in the current study. The rats were housed with food and water *ad libitum* in a temperature-controlled room (23 ± 0.5 °C) with a 12-hour light/dark cycle. From day − 4 to day 0, the rats were treated with either 5-fraction cranial irradiation to a total dose of 20 Gy in 5 days with an XRAD 225Cx (Precision X-Ray) or sham radiation. In brief, rats were anesthetized using 2% isoflurane and placed onto the bed of irradiator. A scout CT was used to center the target region at beam isocenter within the field of view. Then a 2.5 cm collimator was placed over the CT tube and a 0.3 mm copper treatment filter was inserted. In each fraction, a 4 Gy dose was delivered using two opposing lateral beams. The lateral beam times were 31.1 s and 31.7 s with a dose rate of 3.82 Gy/min. The voltage used was 225Kv and the current was set to 20 mA. The sham radiation procedure was designed to closely mimic the conditions experienced by the radiation-treated animals. Rats in the sham radiation group underwent the same preparation and handling as those receiving radiation treatment. They remained on the radiator bed for the exact same duration as their radiation-treated counterparts. However, during this period, the sham group did not receive any actual radiation exposure. This approach controlled for potential confounding factors, such as stress from restraint, anesthesia effects, and environmental conditions, allowing for a more accurate assessment of radiation-specific outcomes.

### Electrophysiology recording in vivo

On designated days after irradiation, rats were anesthetized (urethane 1.3 g/kg i.p. for acute recording; ketamine 90 mg/kg i.p. and 9 xylazine mg/kg i.p. for multi-electrode implantation) and placed in a stereotaxic frame with body temperature maintained at 37 °C by a homoeothermic warming blanket (Harvard Apparatus, USA). Extracellular recordings were then obtained in vivo as described in our previous publications [12, 26]. In brief, glass electrodes (2 M NaCl) were positioned in the target area through a small burr hole in the skull by using coordinates based on the Atlas of Paxinos and Watson (PFC: 3.0 mm anterior to bregma, 0.8 mm lateral to the midline, 3.5–4.0 mm deep; VTA: 3.0 mm anterior to lambda, 0.5–0.9 mm lateral to the midline, 6.5–8.5 mm deep). Dopamine neurons were identified according to published criteria [26, 27, 28], including the presence of a long action potential duration (2–5 ms), a relatively slow firing rate (< 10 Hz) characterized by an regular or burst firing pattern, low pitch sounds produced on an audio amplifier, and a ≥ 1.1 ms duration from the start of the action potential to the negative trough. Multi-electrode arrays were implanted into targeted brain areas to cover the VTA and PFC regions. Small electrode holes were drilled above the target areas, as well as 4 smaller holes around the electrode hole (3 mm away from the edge of the electrode hole). Jeweler’s screws were threaded into the 4 smaller holes as the anchors. Then the electrodes were smoothly lowered into the recording areas with an electronic micromanipulator. A small amount of dental cement was applied around the electrode and screws by using a small spatula. As the cement thickened, it became molded around the screws and electrode to form a smooth cap. The wound was closed with a second layer of dental cement. Animals were returned to housing facilities for a minimum of 2 weeks of recovery before any experiments were conducted. All animals were euthanized after the experiments were completed, their brains removed, and recording sites were verified by visual inspection. Data were analyzed by using NeuroExplorer and GraphPad Prism.

### Immunofluorescence

Rats were induced into a deep state of anesthesia by using isoflurane and subsequently perfused through the ascending aorta with phosphate-buffered saline, followed by cold 4% paraformaldehyde in 0.1 M PBS. The brains were then removed, fixed in 4% paraformaldehyde for 48 h, and cryoprotected through sequential immersion in 20% and then 30% sucrose solutions. Serial frozen sections containing the specified target regions were cut at a thickness of 10 μm by using a cryostat for immunofluorescence analysis. These sectioned brain slices were affixed to glass slides (Southern Biotech). After a 1-hour block in 5% bovine serum albumin and 0.2% Triton X-100 in PBS at room temperature, the sections were incubated overnight at 4 °C in a mixture of 5% BSA and 0.2% Triton X-100 in PBS, which included primary antibodies against tyrosine hydroxylase (TH) (ab6211, Abcam, 1:500). The next day, sections were thoroughly washed and incubated with AlexaFluor secondary antibodies for 1 h at room temperature. TH-positive cells were quantified in randomly selected regions (100 μm × 100 μm) from each brain section. A minimum of 4 sections from both sham- and radiation-treated rats were quantified for each experiment. In every instance, images were captured by using identical acquisition parameters by experimenters blinded to the treatment groups.

### Western blotting

Brain tissue samples were collected from 3 rats in each treatment group that had been deeply anesthetized with isoflurane. The samples were snap-frozen in liquid nitrogen. Tissues were later disrupted in HEPES-sucrose buffer and then centrifuged at 12,000 × *g* at 4 °C for 10 min. The supernatant was then transferred into new tubes and denatured with sample buffer (Laemmli 2× Concentrate, Sigma-Aldrich) for 10 min at 90 °C. Lysates were separated by using SDS-PAGE and transferred to polyvinylidene fluoride membranes (Bio-Rad). After blocking with 5% fat-free milk in TBST buffer for 1 h at room temperature, membranes were incubated with anti-dopamine D2 receptor (ab5084P, Sigma-Aldrich, 1:500) or anti-β-actin (4967 S, Cell Signaling, 1:600) in 5% fat-free milk in TBST buffer overnight at 4 °C. The next day after being washed with TBST, membranes were incubated with secondary antibodies diluted with 5% fat-free milk in TBST for 1 h at room temperature, and the reactive bands were then detected with SuperSignal West Femto Substrate (Themo Scientific).

### Spontaneous alternation Y-Maze tests

Rats underwent a 7-day acclimatization period before experiments to ensure their familiarity with the environment, staff, smells, and noises. The Y-maze was cleaned with ethanol before each test and left to dry completely. Animals were acclimatized to the testing room for 1 h before being placed in a distal maze arm, facing the center. Videos were recorded during an 8-minute exploration, uninterrupted by disturbances.

## Results

### Cranial irradiation transiently alters dopamine neuron firing patterns, but not firing rates, in the VTA

**Fig. 1:**
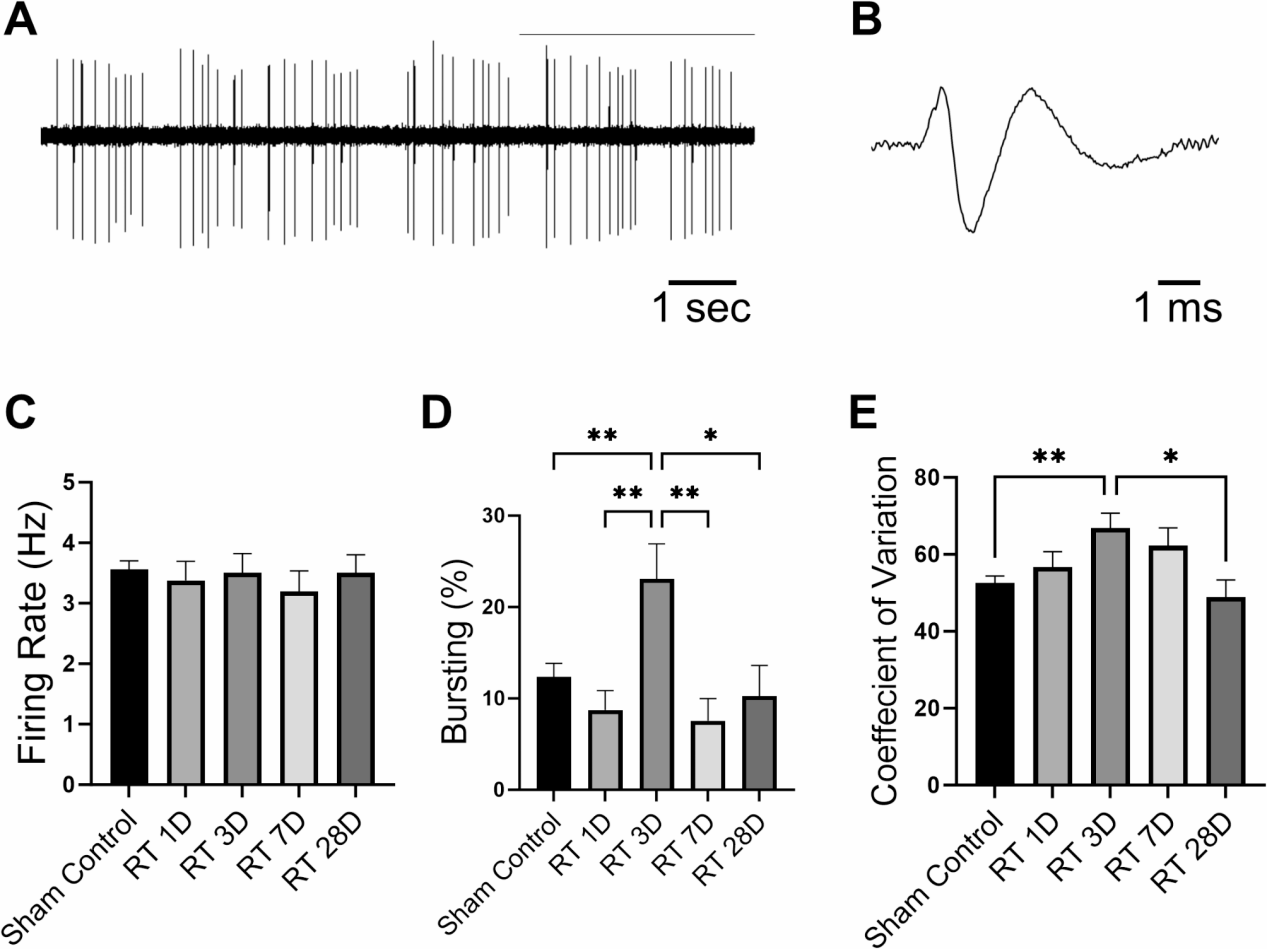
Cranial irradiation induces alterations in the firing pattern of dopamine neurons in the ventral tegmental area (VTA) without affecting their firing rate. In vivo, VTA dopamine neurons display spontaneous activity, generating both single spikes and bursts. Extracellular recordings from VTA dopamine neurons [(**A**) spikes train and (**B**) single action potential)] were obtained to assess (**C**) firing rates, (**D**) bursting levels, and (**E**) the coefficient of variation of interspike intervals at 1, 3, 7, and 28 days after the last of 5 treatment fractions of cranial irradiation to a total dose of 20 Gy. Although no significant changes were observed in VTA dopamine neuron firing rates (sham control: 3.56 ± 0.14 Hz, *n* = 149 from 20 rats; day 1: 3.38 ± 0.32 Hz, *n* = 43 from 5 rats; day 3: 3.50 ± 0.32 Hz, *n* = 44 from 5 rats; day 7: 3.19 ± 0.34 Hz, *n* = 39 from 5 rats; day 28: 3.51 ± 0.29 Hz, *n* = 39 from 5 rats; one-way ANOVA F_(4, 309)_ = 0.318, *P* = 0.866), both bursting levels and coefficient of variation initially increased (**Bursting**: day 3: 23.09 ± 3.84, *n* = 44 from 5 rats; vs. sham control: 12.37 ± 1.48, *n* = 149 from 20 rats; one-way ANOVA, F_(4, 309)_ = 4.727, *P* = 0.001; **CV**: day 3: 66.80 ± 3.88, *n* = 44 from 5 rats; vs. sham control: 52.57 ± 1.82, *n* = 149 from 20 rats; one-way ANOVA, F_(4, 309)_ = 4.153, *P* = 0.003) and then returned to control levels (**Bursting**: day 28: 10.27 ± 3.33, *n* = 44 from 5 rats; vs. sham control: 12.37 ± 1.48, *n* = 149 from 20 rats; one-way ANOVA, F_(4, 309)_ = 4.727, *P* = 0.95; **CV**: day 28: 48.92 ± 4.47, *n* = 44 from 5 rats; vs. sham control: 52.57 ± 1.82, *n* = 149 from 20 rats; one-way ANOVA, F_(4, 309)_ = 4.153, *P* = 0.93). *P < 0.05; **P < 0.01

To investigate the effects of cranial irradiation on the firing activities of VTA dopamine neurons, we used in vivo acute extracellular recordings to collect dopamine neuron firing segments from sham control and irradiated rats at 1, 3, 7, and 28 days after exposure (Fig. 1A and B). Firing rates were not significantly altered in VTA dopamine neurons after cranial radiation to a total dose of 20 Gy administered in 5 fractions over 5 days (Fig. 1C). However, we observed changes in neuron firing patterns, a transient increase in the bursting level of these dopamine neurons after radiation. However, this radiation-induced enhancement in bursting was not sustained and measures returned to the levels observed in the sham control group within 4 weeks (Fig. 1D).

**Fig. 2:**
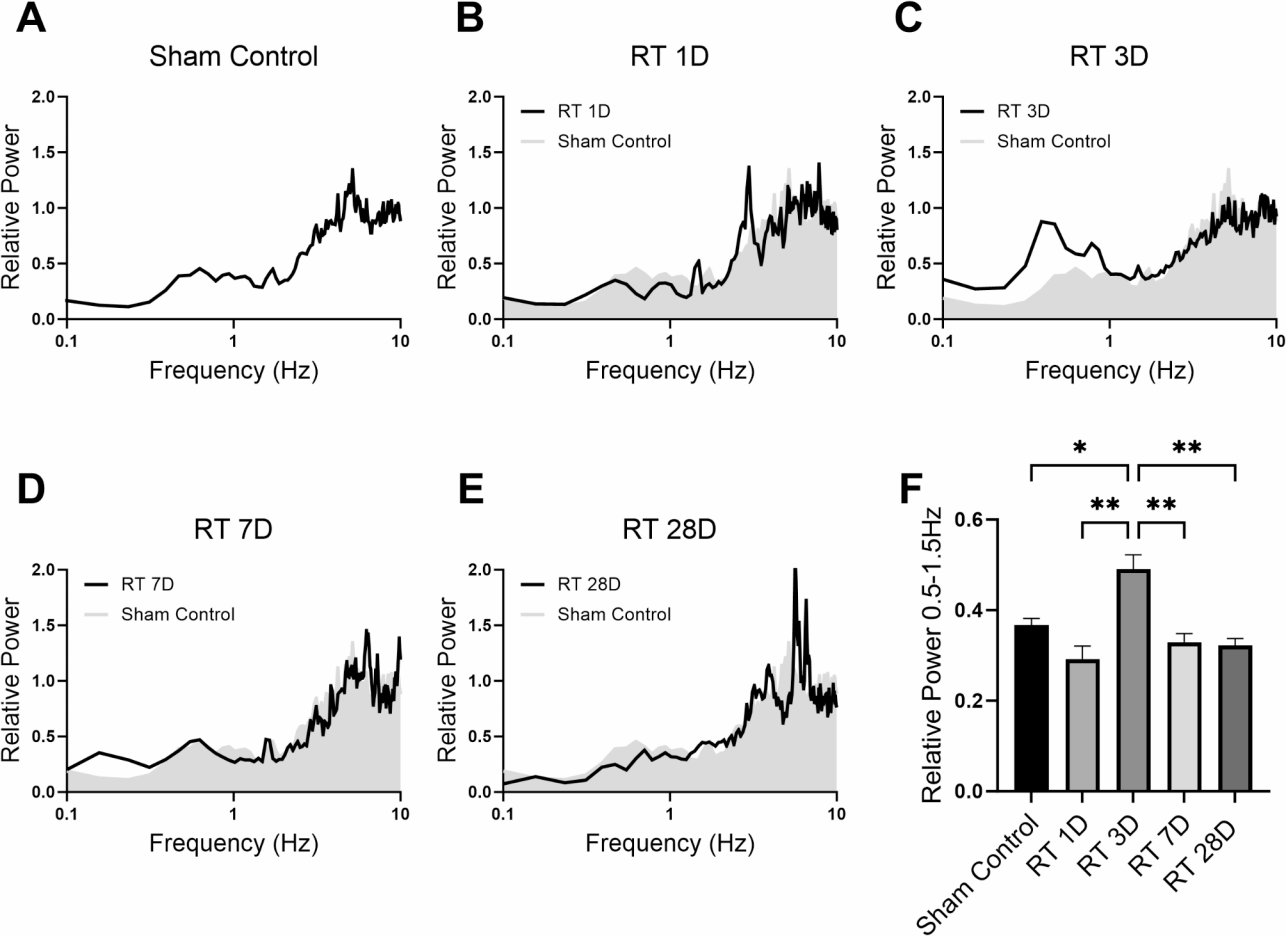
Cranial irradiation dynamically alters the slow oscillatory firing activity of dopamine neurons in the ventral tegmental area (VTA). As a novel concept that complements the traditional firing mode of dopamine neurons, slow oscillations, represented by spectral power between 0.5 Hz and 1.5 Hz, were assessed in (**A**) sham (control)-treated rats or at (**B**) 1 day, (**C**) 3 days, (**D**) 7 days, and (**E**) 28 days after irradiation. Consistent with the finding on bursting and CV, the spectral power of slow oscillation powers initially increased before returning to control levels (**F**, Welch’s ANOVA, F_(4.000,29.40)_ = 6.948, *P* < 0.01; sham control: 0.37 ± 0.01, *n* = 20 rats; day 1: 0.29 ± 0.03, *n* = 5 rats; day 3: 0.49 ± 0.03, *n* = 5 rats, *P* < 0.05 vs. sham control; day 7: 0.33 ± 0.02, *n* = 5 rats; day 28: 0.32 ± 0.01, *n* = 5 rats). *P < 0.05; **P < 0.01

Coefficient of variation (CV) of interspike intervals and spectrum analysis are powerful tools for tracking alterations in neuronal firing patterns. We found a significant increase in the CV of interspike intervals, indicating a notable change in the regularity of neuron firing, on day 3 after cranial radiation (Fig. 1E). These changes were also evident in spectrum analysis, particularly in the spectral powers of slow oscillation within the 0.5–1.5 Hz frequency range (Fig. 2). However, similar to the radiation-induced enhancement in bursting level, the increase in CV values and slow oscillations of neuronal activity were transient, lasting only a few days and returning to control levels within 4 weeks after radiation exposure.

### Cranial irradiation induces prolonged decreases in dopamine neuron density in the VTA

**Fig. 3:**
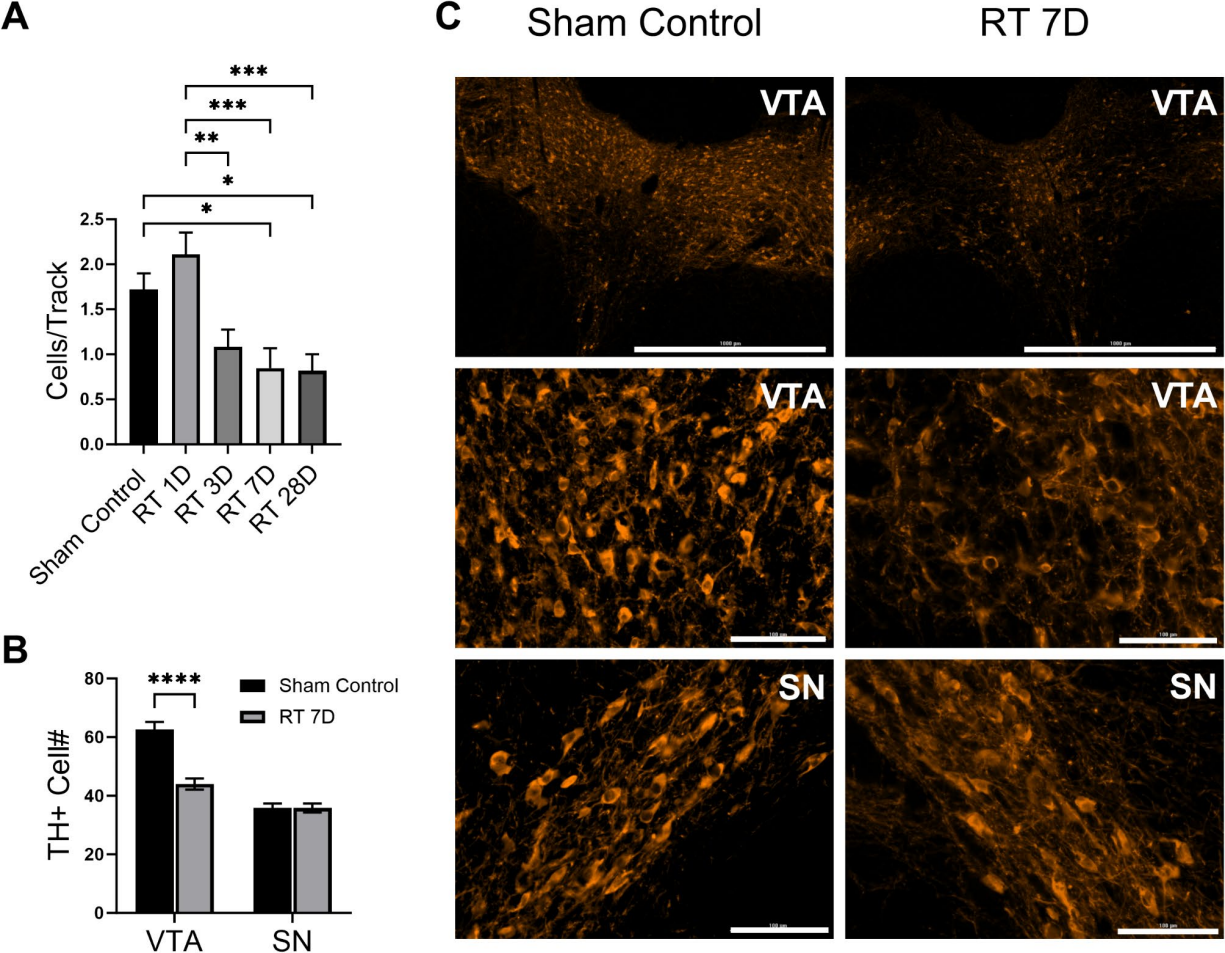
Cranial irradiation prolongedly reduces both the number of dopamine neurons with spontaneous activity and dopamine neuron density within the ventral tegmental area (VTA). (**A**) We used a cells/track technique to evaluate the numbers of dopamine neurons with spontaneous firing at 1 day, 3 days, 7 days, and 28 days after irradiation. The decrease persisted for 4 weeks after radiation exposure (one-way ANOVA, F_(4,67)_ = 7.626, *P* < 0.001; sham control: 1.72 ± 0.17, *n* = 18 tracks; day 1: 2.11 ± 0.24, *n* = 18 tracks; day 3: 1.08 ± 0.19, *n* = 12 tracks; day 7: 0.85 ± 0.22, *n* = 13 tracks, *P* < 0.05 vs. sham control; day 28: 0.82 ± 0.18, *n* = 11 tracks, *P* < 0.05 vs. sham control; 5 rats were used in each groups). (**B** and **C**) Radiation significantly changed the numbers of tyrosine hydroxylase (TH)-positive neurons within VTA (sham control: 62.59 ± 2.55, *n* = 17 slices from 5 rats; day 7: 44.00 ± 1.89, *n* = 12 slices from 5 rats; *t* test *P* < 0.01) but not in the substantia nigra (SN) (sham control: 35.88 ± 1.50, *n* = 16 slices from 5 rats; day 7: 35.83 ± 1.53, *n* = 12 slices from 5 rats; *t* test *P* = 0.98). *P < 0.05; **P < 0.01

By using the cells/track method during recordings, we monitored changes in the number of dopamine neurons exhibiting spontaneous firing. Unlike the transient impact on neuronal firing patterns, we identified a significant and prolonged decrease in the density of spontaneously firing dopamine neurons within the VTA for at least 4 weeks after radiation exposure (Fig. 1A). This implies that a reduced number of dopamine neurons were actively engaged in the neuronal network after irradiation. To further explore the potential causes of neuronal “silent status”, we performed immunohistochemical staining of dopamine neurons with the TH antibody on VTA brain slices. The immunohistochemical images revealed a significant decrease in TH + signal as early as 1 week after radiation, suggesting that cranial radiation induces cell death within VTA dopamine neurons. Interestingly, the density of dopamine neurons in the substantia nigra (SN) remained unaffected by irradiation (Fig. 3B and C).

### Cranial irradiation dynamically alters D2 dopamine receptor functions

**Fig. 4:**
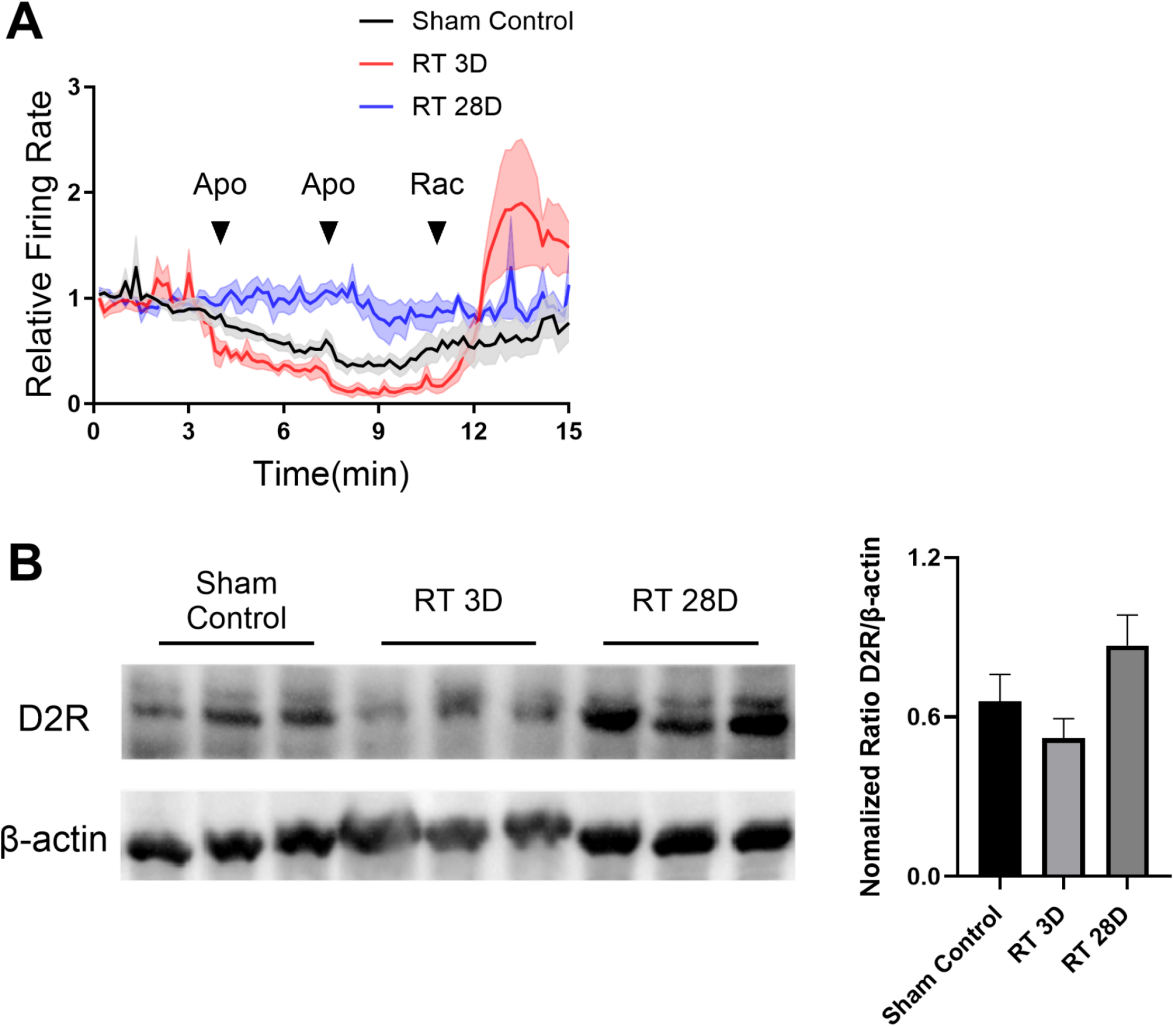
Cranial irradiation disrupts the function of D2 receptors without significantly altering their density within the prefrontal cortex (PFC). (**A**) The effects of radiation exposure are evident in the altered response of ventral tegmental area (VTA) dopamine neurons to apomorphine (D2 receptor agonist) and raclopride (D2 receptor antagonist), exhibiting a transient pattern of hypersensitization followed by desensitization of D2 receptors (solid line: mean; shadow fills: SEM; *n* = 4 rats in each group). (**B**) Western blotting results revealed a trend of dynamic changes in D2 receptor expression in the PFC following radiation exposure; however, one-way ANOVA did not detect any significant differences (F_(2,6)_ = 3.196, *P* = 0.114)

Give the pivotal role of dopamine D2 receptor in regulating the firing activities of dopamine neurons, we sought to investigate the effect of cranial irradiation on D2 receptors. In this study, we conducted experiments involving the administration of apomorphine (a D2 receptor agonist, 50 µg/kg i.v.) and raclopride (a D2 receptor antagonist, 100 µg/kg i.v.) during neuronal recordings. Our findings indicated a transient hypersensitization of D2 receptors, followed by a prolonged desensitization. Specifically, at day 3 after radiation exposure, the apomorphine-induced inhibition of firing rates in dopamine neurons was significantly augmented. Interestingly, administration of the D2 receptor antagonist raclopride at the same dose not only restored firing rates to baseline levels observed in the sham control group but also elicited an increase to higher levels. However, this acute hypersensitization was not sustained. By 4 weeks after radiation exposure, the firing rates of dopamine neurons no longer responded to challenges with either apomorphine or raclopride. This suggests a complete desensitization of D2 receptors induced by cranial irradiation (Fig. 4A).

We previously explored the effects of cranial radiation on the PFC, a brain region receives dopaminergic inputs from the VTA as part of the mesocortical neural network, responsible for mediating various cognitive aspects. Although western blot results indicated a trend toward changes in D2 receptor expression, no significant alterations were observed in the PFC following radiation exposure. (Fig. 4B). This evidence suggests that cranial radiation dynamically affects the functional aspects of D2 receptors over time, without significantly impacting receptor density.

### Cranial irradiation inhibits functional coupling between PFC and VTA

**Fig. 5:**
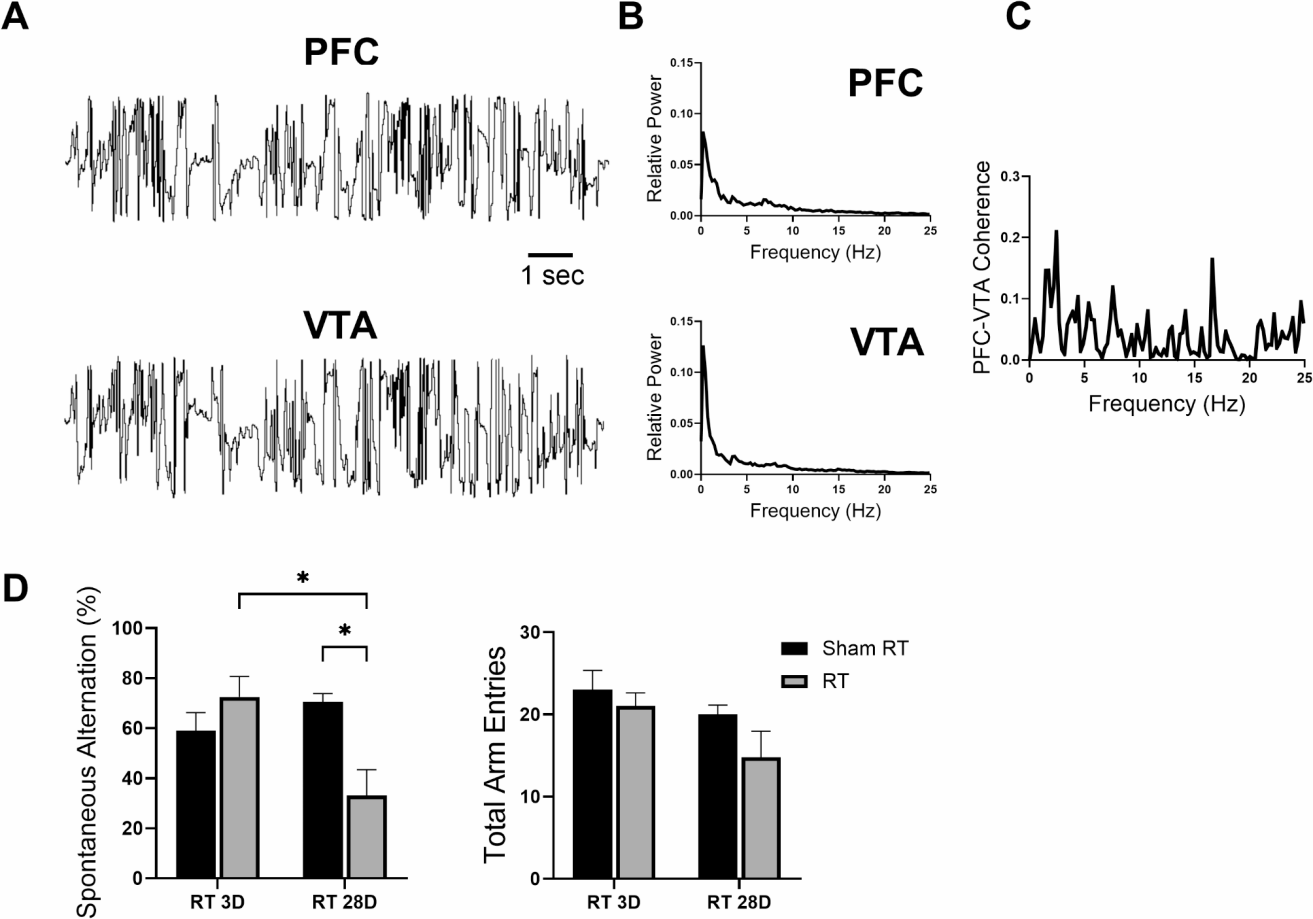
Coupling between firing activities in the ventral tegmental area (VTA) and prefrontal cortex (PFC). (**A**) Segments of local field potentials recorded simultaneously from the PFC and VTA in a free-moving animal, as visualized with an implanted multi-electrode array. (**B**) The autospectrum of 2-min local field potentials recorded simultaneously from the PFC and VTA. (**C**) The coherence spectrum between the two recordings. (**D**) Histograms of Y-maze spontaneous alternation indicated a significant decrease in spontaneous alternation rates in rats 28 days after irradiation, compared to the sham control group (*n* = 5, two-way ANOVA, F_(1,16)_ = 10.94, *P* = 0.0045; Sham RT vs. RT on day 28 post irradiation, *P* = 0.016, *P < 0.05), while there was no statistical significance between groups in the total arm entries (*n* = 5, two-way ANOVA, F_(1,16)_ = 0.52, *P* = 0.48)

**Fig. 6:**
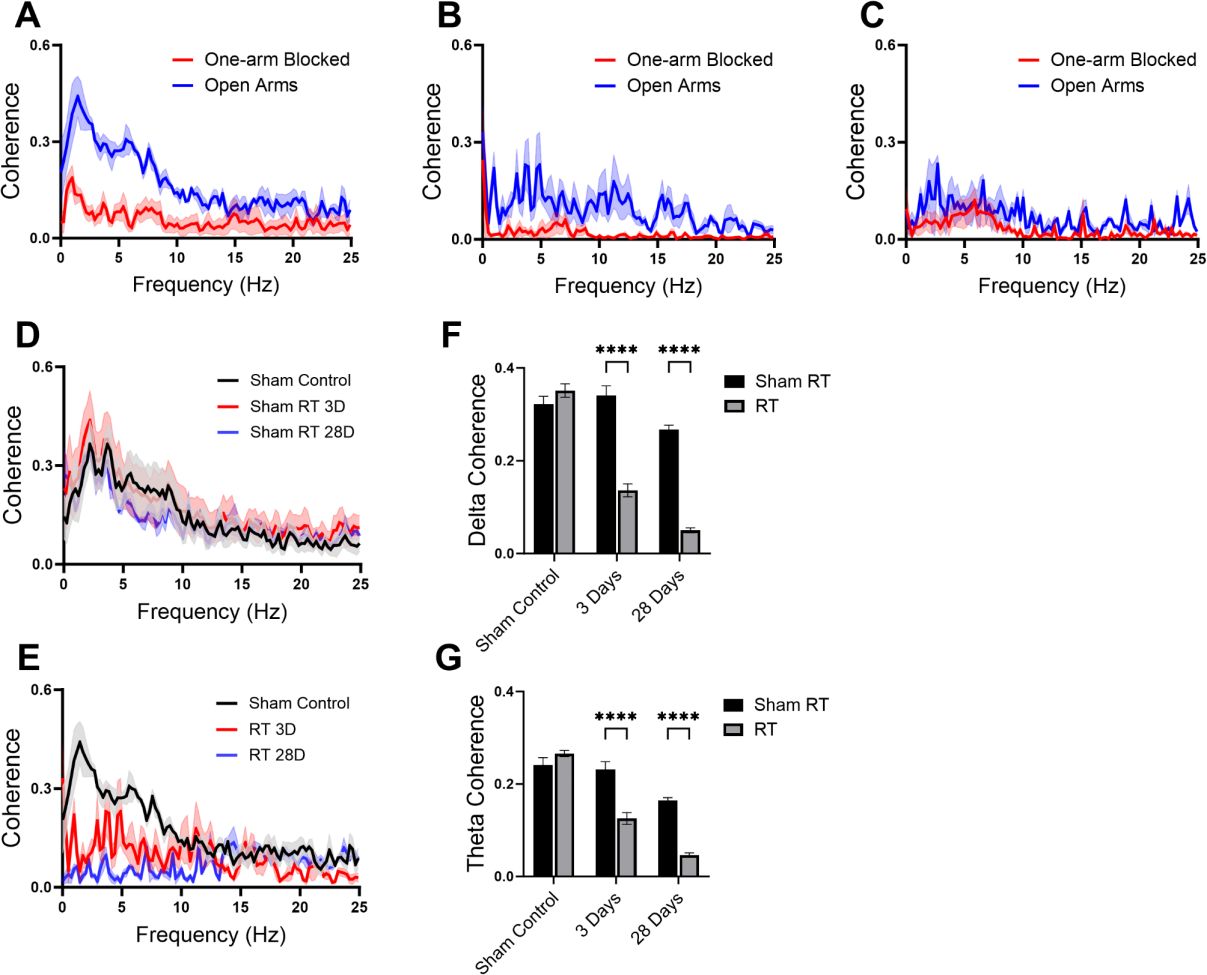
Cranial radiation impairs coupling between the prefrontal cortex (PFC) and ventral tegmental area (VTA). Local field potentials were recorded simultaneously from the PFC and VTA in free-moving animals during spontaneous alternating Y-maze tests before and after radiation treatments. (**A**) Significant distinctions were noted in the slow-frequency bands (Delta and Theta) of coherence between the PFC and VTA during one-arm-blocked and open-arms Y maze tests. (**B** and **C**) The robust coherence observed in slow-frequency bands of the PFC-VTA showed sustained depression at both day 3 (**B**) and day 28 (**C**) after irradiation (solid line: mean; shadow fills: SEM; *n* = 3 rats in each group). (**D** and **E**) Differing from the sham treatment (**D**), cranial radiation (**E**) substantially disrupted the functional coupling between the PFC and VTA, manifesting as an extended suppression in coherence between these two brain regions within (**F**) Delta bands (two-way ANOVA, F_(2, 246)_ = 45.01, *P* < 0.01; RT3D 0.14 ± 0.01 vs. sham3D 0.34 ± 0.01, *P* < 0.01; RT4W 0.05 ± 0.00 vs. sham4W 0.27 ± 0.01, *P* < 0.01, 3 rats in each group) and (**G**) Theta bands (two-way ANOVA F_(2, 282)_ = 22.68, *P* < 0.01; RT3D 0.13 ± 0.01 vs. sham3D 0.23 ± 0.01, *P* < 0.01; RT4W 0.05 ± 0.00 vs. sham4W 0.16 ± 0.00, *P* < 0.01, 3 rats in each group). **P < 0.01

The use of electrode implantation in freely moving animals has provided a pivotal bridge between electrophysiological recordings and behavioral assessments. One of the core facets of our study involved the simultaneous collection of electrophysiological signals from two key brain regions within mesocortical pathways, the PFC and the VTA, in rats subjected to spontaneous alternation Y-maze tests (Fig. 5). Our results demonstrated that the spontaneous alternation rates were significantly decreased in radiated rats at day 28 post irradiation. Although there was also a trend toward a decrease in the total number of arm entries in this group, this difference did not reach statistical significance (Fig. 5D). In addition to radiation-induced behavioral impairment in spatial working memory and exploratory behavior observed in animals, which is consistent with prior reports [29, 30], we identified a profound transformation in the slow oscillation coherence between local field potentials within the PFC and VTA, specifically within the 1–10 Hz frequency band, a functional coupling closely linked to the rats’ choice of entering the arms of the maze. Our findings exhibited a consistent pattern in the trial when one of the three arms of the Y-maze was obstructed: a marked difference was observed in the slow frequency segment of coherence between PFC and VTA signals compared with the scenario where all arms of Y-maze were accessible (Fig. 6A-C), highlighting the critical role of PFC-VTA coupling, particularly within the delta and theta EEG bands, in shaping spatial working memory.

Our study also revealed that the coherence of PFC-VTA signals within the delta and theta EEG bands was notably susceptible to the influence of cranial radiation. Notably, the divergence between the irradiated group and the sham controls became evident as early as 3 days after radiation. Although PFC-VTA coherence also showed a trend toward a slow decrease by 4 weeks after irradiation in sham controls, the depression in the Delta and Theta bands persisted, suggesting a lasting alteration in the functional connectivity between the VTA and PFC within the mesocortical network (Fig. 6D-G).

## Discussion

We identified a substantial and previously unknown effect of cranial irradiation on the mesocortical dopaminergic pathway. We found significant alterations in the firing patterns of VTA dopamine neurons, accompanied with a notable reduction in the overall density of VTA dopamine neurons, as well as a decrease in the number of dopamine neurons exhibiting spontaneous firing. In addition, irradiation led to a dynamic alteration and prolonged desensitization of D2 receptors. We further used implanted multi-electrode arrays and discerned a distinct inhibitory effect of irradiation on functional coupling between the VTA and PFC regions in free-moving rats performing behavioral tasks. For the first time, our findings collectively constitute the initial direct evidence of impaired midbrain dopamine system functionality after radiation exposure. Specifically, our results delineate the acute and prolonged effects of radiation on the mesocortical dopaminergic pathway in several aspects, from alterations in receptor functions to disruptions in coupling between distinct brain regions.

The mesocortical dopaminergic pathway, characterized by dopaminergic projections originating from the VTA to the PFC, is recognized for its pivotal neuro-modulatory influence on mnemonic functions mediated by the frontal lobes. Pioneering work such as the seminal study by Brozowki et al. in early 1979 revealed that depleting dopamine in the PFC of primates resulted in impairments on delayed response tasks comparable to those observed after the complete removal of the frontal lobes [31]. Since then, a substantial body of research has been dedicated to investigating the functional role of mesocortical dopamine in cognitive functions, as well as identifying the specific dopamine receptor subtypes through which these actions are mediated. Although the precise mechanism by which mesocortical dopamine regulates cognitive function remains unclear, disruptions in dopaminergic function have been consistently observed in various brain diseases associated with cognitive impairments [14, 17, 20, 23]. 

As a crucial neuromodulator within the mesocortical network, dopamine release is subject to various regulatory factors, with action potentials emerging as a prominent regulator among them. In microdialysis studies, the introduction of tetrodotoxin into the striatum through reverse dialysis effectively impedes action potential firing, leading to a substantial reduction of approximately 70% in extracellular dopamine levels [32]. Although our study revealed no significant alteration in the firing rate of VTA dopamine neurons, their firing patterns exhibited significant changes after exposure to cranial radiation. Notably, axonal dopamine release does not scale linearly with action potential firing, and not all action potentials result in the same level of dopamine release [33, 34]. Traditionally, dopamine neuron firing patterns take place in one of two modes: single spiking and bursting [35, 36]. In vivo recordings of spontaneously active dopamine neurons commonly reveal a combination of both firing modes, with the extent of bursting varying significantly from cell to cell. Furthermore, within a given dopamine neuron, the level of bursting can be dynamically altered in response to various information-processing mechanisms [37]. The rapid firing of dopamine neurons can result in a substantial release of dopamine. Earlier research has demonstrated that stimulating dopamine neurons in a burst pattern produces a more substantial dopamine release compared with tonic stimulation, even with a consistent number of impulses per second and total impulses [34, 38]. Besides the classic bursting criteria, recent advances in understanding dopamine neuron firing patterns include the recognition of the slow-oscillatory concept, complementing the traditional two-firing modes [39, 40]. It is believed that by altering their firing patterns, these dopamine neurons process and encode information, regulating their impact on postsynaptic neurons through rhythmic dopamine release. Influential theories posit that these phasic dopamine releases, which could underlie changes in synaptic plasticity, function as teaching signals in reinforcement learning and motivational signals in reward processing [41]. In our study, we assessed changes in VTA dopamine neuron firing patterns through three distinct approaches: classic bursting level, slow-oscillation spectral power, and the CV in interspike intervals. All three methods yielded consistent results, indicating that cranial irradiation transiently but significantly altered the firing pattern of dopamine neurons, which may potentially trigger a short but robust release of dopamine after exposure. Although our findings indicated that firing patterns eventually returned to the control level in 4 weeks after irradiation, it is plausible that lasting changes were initiated within the mesocortical network. In neuroscience, synaptic plasticity refers to the ability of synapses to strengthen or weaken over time in response to changes in activity levels. Memories are thought to be encoded within complex, interconnected neural circuits, making synaptic plasticity a crucial neurochemical foundation for learning and memory. Plastic changes typically arise from structural and functional alterations at the synapse. Several mechanisms contribute to synaptic plasticity, such as changes in the amount of neurotransmitter released. This suggests that the short but strong “dopamine surge” immediately following radiation exposure could induce long-lasting changes in synaptic plasticity and signaling within these neural networks, ultimately affecting cognitive functions such as memory and learning.

Another long-lasting alteration resulted from cranial radiation is the population of activated dopamine neurons. Notably, not all dopamine neurons exhibit spontaneous firing, both in vivo and ex vivo. Previous studies proposed that, under baseline conditions, more than half of VTA dopamine neurons are classified as “silent cells.” These dormant neurons can be activated by external stimuli. As the neurons adapt to changes in their functions, the proportion of spontaneously active cells may be influenced by drugs, treatments, or pathological developments [42, 43, 44, 45]. Grace and colleagues introduced a method to assess the ratio of active to silent cells, known as “population activity.” This involves tallying the number of cells detected each time the electrode is lowered within the recording region, a technique requiring expertise in recording [45]. By applying this approach, we showed that cranial radiation exposure significantly reduced the population of VTA dopamine neurons exhibiting spontaneous firing for an extended duration. Moreover, the density of dopamine neurons within the VTA was significantly decreased, as assessed by TH staining. This evidence suggests a prolonged fundamental shift in the baseline status of VTA dopamine neurons after radiation exposure, potentially influencing information processing capabilities and contributing to dysfunction within the mesocortical pathway.

Accumulating evidence suggests that dopamine influences cognition, with the specific cognitive effects being contingent upon the subtypes of dopamine receptor activated [46, 47]. Although most studies have focused on D1 receptors, recent attention has turned towards the potential role of D2 receptors. Beyond its established role in regulating dopamine neuron firing activities, the D2 receptor is increasingly recognized for its involvement in modulating diverse cognitive processes such as attention, working memory, executive functions, and learning [48]. Research indicates that disruption in dopamine D2 receptor function or availability may be associated with cognitive disorders like schizophrenia [49], attention deficit hyperactivity disorder [50], stress [51], and other conditions affecting cognitive function. Medications targeting dopamine receptors, including D2 receptors, are occasionally used in treating these disorders to modulate dopamine activity and ameliorate cognitive symptoms. In our prior research, we demonstrated the neuroprotective capabilities of memantine against radiation-induced neuronal toxicity [11, 52]. Clinical evidence in adult patients further supports the partial protective effects of memantine on radiation-induced cognitive deficits [53]. Notably, memantine functions not only as a glutaminergic NMDA receptor antagonist but also as a dopamine D2 receptor agonist [54], which may protect D2 receptors from radiation-induced desensitization. In the present study, we report, for the first time, alterations in D2 receptors after cranial radiation exposure. Although no significant changes were found in the levels of D2 receptors in the PFC region, an extended desensitization of D2 receptors was observed after irradiation. This prolonged desensitization could significantly impair the regulation of dopamine neuron firing properties and dopamine release patterns, disrupting neuronal signal transmission and potentially leading to dysfunction within the mesocortical network.

One of the most significant findings of our study is that we could, by using implanted multielectrode arrays in free-moving animals, directly evaluate the effects of radiation on functional coupling between the VTA and PFC during behavioral tasks. In 2000, Braver and Cohen proposed that the PFC functions to maintain task-relevant information through a dopamine-dependent manner [55]. According to this model, coordinated activation of the dopamine system is required to enable proper neuronal ensembles in the PFC to selectively amplify salient information. Dopamine within the PFC may have a dual role in modulating the effectiveness of cognitive functions including working memory and attention. Initially, dopamine has the capacity to regulate the gain of specific neuron classes; for instance, PFC pyramidal neurons can respond to dopamine by enhancing single-neuron gain, optimizing signal-to-noise ratios and thereby increasing perceptual sensitivity [56]. In addition, dopamine can facilitate the existence of two stable states within individual pyramidal neurons– a resting state and sustained activity. This bi-stability is theorized to form a neural basis for the establishment of working memory, specifically short-term memory, by actively maintaining signals (such as perceptual input or recalled memories) during periods of retention [57, 58]. 

Undoubtedly, the compromised functionality of VTA dopamine neurons observed in this study likely disrupts connectivity between the VTA and PFC. Delta and theta EEG bands, well-established for their critical roles in cognitive processing, have been extensively studied, with dysregulation reported in both clinical and experimental models of cognitive impairment [59, 60, 61, 62]. our current research offers direct evidence that cranial radiation significantly diminishes the functional connectivity in both delta and theta bands between the VTA and PFC. Notably, this disruption correlates with behavioral impairments observed in the Y-maze test, where irradiated rats exhibited significantly reduced spontaneous alternation rates, indicating deficits in spatial working memory and exploratory behavior. This novel insight helps elucidate the mechanisms underlying radiation-induced cognitive decline.

While our study revealed both acute and prolonged effects of radiation exposure, the long-term trajectory of these changes remains unclear. Longitudinal studies with extended observation periods could provide deeper insights into the persistence and potential reversibility of radiation-induced neurobiological alterations. Further research should also explore potential therapeutic interventions, like pharmacological agents targeting dopamine receptors or neuroprotective strategies, to mitigate cognitive deficits linked to cranial irradiation. Addressing these gaps will enhance our understanding of radiation-induced cognitive impairments and inform effective prevention and treatment strategies.

## Conclusion

In conclusion, our study reveals a profound and prolonged suppression of VTA dopamine neuronal function after cranial irradiation that significantly affects the functional connectivity between the VTA and PFC, particularly in the delta and theta bands. These findings offer insight into the mechanisms underlying radiation-induced cognitive deficits. As we expand our understanding of these neural alterations, potential avenues for targeted interventions may emerge, paving the way for advances in mitigating cognitive impairments associated with cranial radiation.

## Electronic supplementary material

Below is the link to the electronic supplementary material.

Supplementary Material 1

Supplementary Material 2

Supplementary Material 3

Supplementary Material 4

## Data Availability

All data from the current study can be made available from the corresponding author upon reasonable request.

## Abbreviations

VTA: Ventral tegmental area
PFC: Prefrontal cortex
RT: Radiation therapy
TH: Tyrosine hydroxylase
CV: Coefficient of variation
SN: Substantia nigra
