# Supplementary figures and images for: Cranial radiation disrupts dopaminergic signaling and connectivity in the mammalian brain
Source: Acta Neuropathol Commun. 2025 Mar 13;13:59. doi: 10.1186/s40478-025-01976-3 (PMC11905640; doi:10.1186/s40478-025-01976-3)

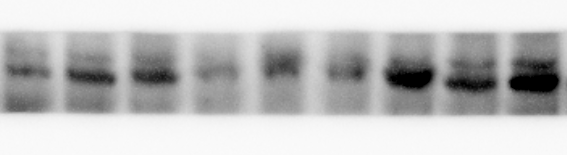

Supplement: Supplementary file 1 — Supplementary Material 1 [file 40478_2025_1976_MOESM1_ESM.tif]

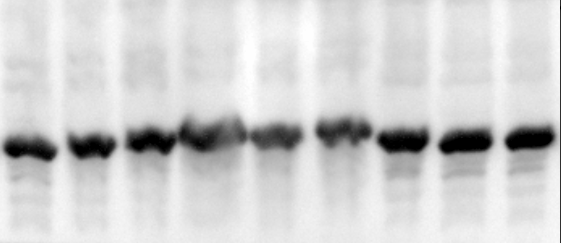

Supplement: Supplementary file 2 — Supplementary Material 2 [file 40478_2025_1976_MOESM2_ESM.tif]

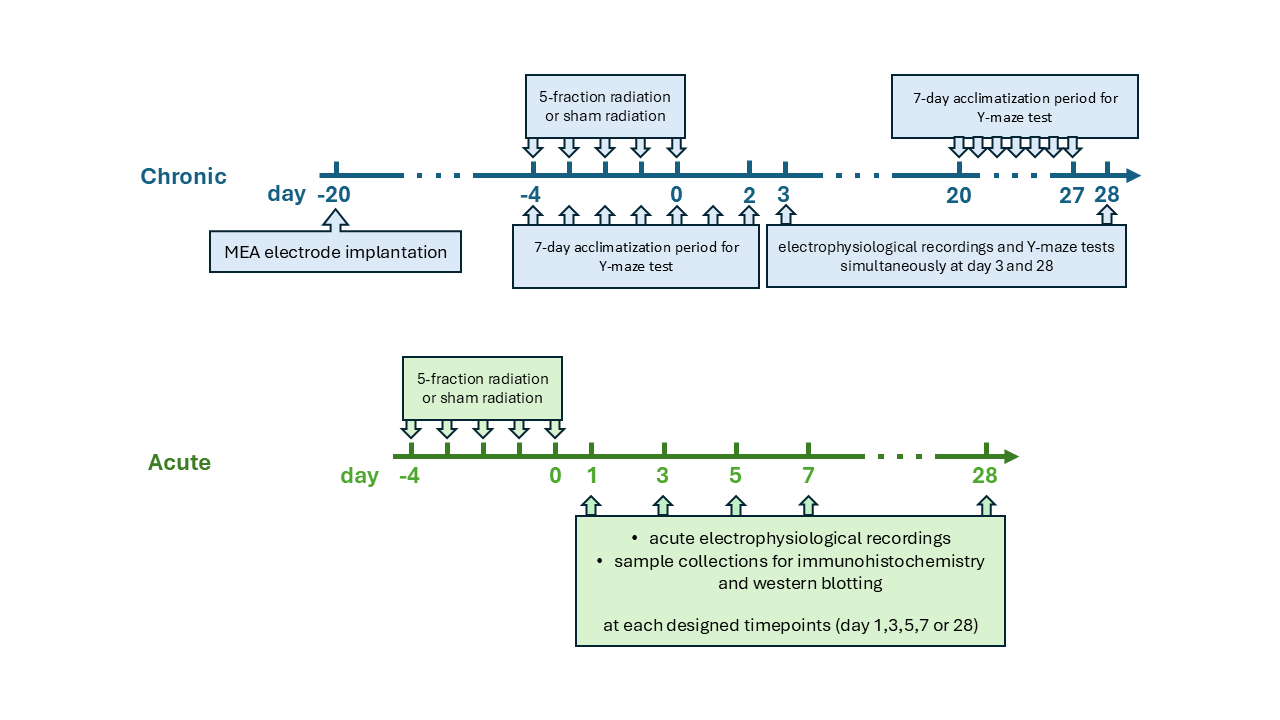

Supplement: Supplementary file 4 — Supplementary Material 4 [file 40478_2025_1976_MOESM4_ESM.tif]
