# Supplementary material for: Cranial radiation disrupts dopaminergic signaling and connectivity in the mammalian brain
Source: Acta Neuropathol Commun. 2025 Mar 13;13:59. doi: 10.1186/s40478-025-01976-3 (PMC11905640; doi:10.1186/s40478-025-01976-3)
Supplement: Supplementary file 3 — Supplementary Material 3 [file 40478_2025_1976_MOESM3_ESM.docx]

Supplemental Figure 1: A schematic representation of the experimental workflow in a temporal sequence.
